# Detection and Quantification of *Nocardia crassostreae*, an Emerging Pathogen, in *Mytilus galloprovincialis* in the Mediterranean Sea Using Droplet Digital PCR

**DOI:** 10.3390/pathogens12080994

**Published:** 2023-07-28

**Authors:** Anna Cutarelli, Francesca Carella, Francesca De Falco, Bianca Cuccaro, Fabio Di Nocera, Donatella Nava, Gionata De Vico, Sante Roperto

**Affiliations:** 1 Istituto Zooprofilattico Sperimentale del Mezzogiorno, 80055 Portici, Italy; 2 Dipartimento di Biologia, Università degli Studi di Napoli Federico II, 80126 Napoli, Italy; 3 Dipartimento di Medicina Veterinaria e Produzioni Animali, Università degli Studi di Napoli Federico II, 80137 Napoli, Italy

**Keywords:** *Nocardia crassostreae*, mussel, *Mytilus galloprovincialis*, Mediterranean Sea, droplet digital PCR (ddPCR), real-time qPCR

## Abstract

*Nocardia crassostreae* is a novel pathogen responsible for infections in oysters (*Crassostrea gigas*) and mussels (*Mytilus galloprovincialis*). *N. crassostreae* is also responsible for nocardiosis both in immunocompetent and immunocompromised patients. We investigated *N. crassostreae* DNA in mussels grown in marine sites of the Mediterranean Sea in the Campania Region. We examined 185 mussel pooled samples by droplet digital PCR (ddPCR) and real-time quantitative PCR (qPCR), each pool composed of 10 mussels and 149 individual mussels. ddPCR detected *N. crassostreae* DNA in 48 mussel pooled samples and in 23 individual mussel samples. qPCR detected *N. crassostreae* DNA in six pooled samples and six individual mussel samples. The two molecular assays for the detection of *N. crassostreae* DNA showed significant differences both in the pooled and in individual samples. Our study demonstrated that ddPCR outperformed real-time qPCR for *N. crassostreae* DNA detection, thus confirming that ddPCR technology can identify the pathogens in many infectious diseases with high sensitivity and specificity. Furthermore, in individual mussels showing histological lesions due to *N. crassostreae*, the lowest copy number/microliter detected by ddPCR of this pathogen was 0.3, which suggests that this dose could be enough to cause infections of *N. crassostreae* in mussels.

## 1. Introduction

The genus *Nocardia* encompasses several species of Gram-positive, filamentous bacteria that cause clinical diseases in animals, including humans [1]. To date, 115 species of *Nocardia* have been recognized. Recently, an additional 28 potentially novel species have been identified [2]. *Nocardiae* are ubiquitous organisms distributed worldwide in the environment as saprophytic components of fresh and salt water, soil, dust, decaying vegetation, and decaying fecal deposits from animals. Some species have a territorial prevalence as their distribution can be modulated by a specific climate [3]. Most nocardial infections in humans are caused by *N. asteroides*, which is prevalently responsible for pulmonary infections. These infections can remain localized or spread to other organs via the lymphohematogenous pathway. Nocardial infections as cutaneous diseases result from hematogenous dissemination or, rarely, traumatic percutaneous implantations [4]. *Nocardia* infections often affect immunocompromised individuals. However, approximately one-third of infections occur in immunocompetent patients, which suggests that the immune system is not the only factor influencing the risk of infection [5].

Nocardiosis is a rare infection that has gained attention due to its increased rate of occurrence in recent years [5]. While the incidence of nocardial disease is increasing, there is a debate about whether *Nocardiae* could be considered emerging pathogens [4]. It has been suggested that some *Nocardia* species, such as *N. cyriacigeorgica* and *N. crassostreae*, are true emerging pathogens [6,7,8]. However, the lack of clinical, epidemiological, and laboratory data undermines the significance of *Nocardia* spp. as a potential pathogen [4]. Sometimes, the diagnosis only happens postmortem, highlighting the importance and necessity of improving the diagnostic methodologies [3]. It has been suggested that advances in rapid molecular diagnostic technology will soon place *Nocardiae* in the “extended pantheon of medically important pathogens” [9]. Therefore, the development of PCR-based diagnostic assays for the early diagnosis of infection should be considered a priority for this pathogen [5].

*Nocardia* infections have been reported in both terrestrial and aquatic animals. As far as aquatic animals are concerned, in 1998, *N. crassostreae* was identified as the causal agent of nocardiosis in Pacific oysters (*Crassostrea gigas*) grown in several locations in the Pacific Northwest of the USA and British Columbia, Canada [1]. The disease was called Pacific oyster nocardiosis (PON). In 2008, *N. crassostreae* was isolated in Europe from Pacific oysters grown in Lake Grevelingen, the Netherlands [10]. More recently, *N. crassostreae* was also identified in *Ostrea edulis* and, for the first time, was detected in *Mytilus galloprovincialis*, believed to be a new host, from the Mediterranean Sea, Italy [11].

Droplet digital polymerase chain reaction (ddPCR) is an improved method of conventional PCR that offers greater accuracy and reproducibility than real-time quantitative PCR (qPCR). ddPCR is, at this time, the most-accurate and -sensitive method for quantifying nucleic acids of interest, particularly in cases of low pathogen loads [12]. ddPCR is based on dilution and partitioning of the sample into thousands of nanoliter droplets, whereas molecular analysis by qPCR depends on the standard curve and the quantification cycle (Cq) value. It has been shown that sample partitioning improves the precision and sensitivity of ddPCR versus qPCR [13]. 

The present study aimed to carry out a molecular survey of the presence of *N. crassostreae* by detecting and quantifying its DNA in *Mytilus galloprovincialis* from the Mediterranean Sea of the Gulf of Naples. Furthermore, the diagnostic value of ddPCR, showing the potential advantages of this molecular technology in comparison with real-time qPCR in the diagnosis and epidemiology of nocardiosis, was evaluated.

## 2. Materials and Methods

### 2.1. Samples and DNA Extraction

Mussels (*Mytilus galloprovincialis*) were sampled from raft culture farms in several locations along the Campania Region’s coastline, Bay of Naples, at 15 sites. DNA was extracted from the hepatopancreas samples using NUCLISENS^®^ EASYMAG^®^ (Biomerìeux, ARA, FR) according to UNI EN ISO 15216-1-2021. Each sample was composed of the hepatopancreas from 10 mussels randomly grouped into 185 pools. Furthermore, DNA was extracted from one-sided pooled tissues of 149 sagittally sectioned, individual mussels using the QIAmp DNA Mini Kit (Qiagen, Wilmington, DE, USA). Samples were collected on several dates from April 2021 to January 2022 and stored at −80 °C. The extracted DNAs were quantified by Nanodrop (Thermo Scientific, Waltham, MA, USA).

### 2.2. qPCR

The 16S–23S rRNA intergenic spacer (ITS) region was used to discriminate between different species of Nocardia [14]. Using the Realtime qPCR Assay Entry online web interface from IDT (https://eu.idtdna.com/scitools/Applications/RealTimePCR/, accessed on 30 May 2023), the following primers and probes were designed for *Nocardia crassostreae* (GenBank: AF536419.1) and used for both ddPCR and qPCR: forward, 5′-ACCGTTTGAGTCCTCGAATG-3′; reverse, 5′-TGCCTGGAAAGAACGTCTG-3′; probe (FAM): AGTGTTCCACCCATGAGCGTCC (amplicon length: 126 bp). Primers and probes were ordered as a mix with a primer-to-probe ratio of 3.6 (final concentration of 900 nM of each primer and 250 nM of probe). To perform the LoD and then set up the qPCR, a positive control from the National Reference Laboratory (NRL) for Fish, Shellfish and Crustacean Diseases (Netherlands) was used. The qPCR method has been described previously [15].

### 2.3. ddPCR

For ddPCR, Bio-Rad’s QX100 ddPCR System was used according to the manufacturer’s instructions (https://www.youtube.com/watch?v=Qwma-1Ek-Y4, accessed on 30 May 2023). The details of this procedure have been described previously [16]. Each sample was analyzed in duplicate. Samples with very few positive droplets were reanalyzed to ensure that these low-copy-number samples were not due to cross-contamination. The same samples used as positive controls for qPCR were also tested using ddPCR.

#### Optimization of the ddPCR and qPCR Protocols

To optimize the primer and probe concentrations, 150 nM, 200 nM, and 250 nM for the probes and 500 nM, 750 nM, and 900 nM for the primers were investigated in duplicates using the positive control. As far as ddPCR is concerned, the evaluation took into account: extent of rain, resolution, and amount of fluorescence, whereas the concentration for each primer that generated the lowest cycle threshold (Ct) value was evaluated for qPCR. A 900 nM primer and 250 nM probe mixture was chosen. The thermal gradient was tested in a 55–62 °C range to determine the optimal hybridization temperature of the assay, which, ultimately, resulted in 60 °C. The specificity of the primer and probe mixture of this study was evaluated using *N. asteroides* DNA featured by the lack of positive droplet emission in any case. The repeatability of ddPCR and qPCR was determined by five different DNA concentrations in samples of *N. crassostreae*, evaluating them in four replicates. Finally, the ddPCR and qPCR assays were performed over several days by different operators.

The sensitivity of each assay was calculated as the detection of a minimal bacterial load: limit of detection (LOD). The *N. crassostreae* DNA was detected using qPCR and ddPCR as reported previously [16]. In qPCR, linear regression was used to determine the line of the best fit for the relationship between Ct and bacterium copies. A Ct value of 40 was set as the minimum amount of bacterium copies detected by qPCR. Values <1 copy/μL indicated high sensitivity of the ddPCR protocol according to other authors [16,17]. Supplemental Figure S1 shows the workflow of ddPCR. 

### 2.4. PCR

Twelve of the real-time PCR- and ddPCR-positive samples were amplified by PCR with the same primers used for qPCR. Then, the amplicons were sequenced. To the PCR mixture composed of a 1 µM final concentration of each primer and a PCR buffer (EconoTaq^®^ PLUS 2X Master Mix) (Lucigen, Middleton, WI, USA), 5 μL of template DNA (100 ng) was added to obtain a 25 μL final volume. The thermal profile of the PCR was an initial denaturation at 94 °C for 2 min followed by 35 cycles at 94 °C for 30 s, 60 °C for 30 s, and 72 °C for 15 s and a final extension at 72 °C for 10 min with a Simpli Amp thermocycler (Applied Biosystems, Foster City, CA, USA).

### 2.5. Sequence Analysis

The method for sequence analysis has been described previously [16]. Electropherograms were analyzed using Sequencing Analysis Software v 6.0 (Applied Biosystems, Foster City, CA, USA). The obtained sequences were compared to other sequences in GenBank using BLAST.

### 2.6. Gross and Microscopic Findings

Gross and histological patterns were studied on the 149 individual bivalves. Accordingly, the mussels were dissected: the shells were opened by cutting the adductor muscle, followed by the removal of the tissue mass, then a sagittal section comprising the mantle, gonad, digestive gland, and gills was taken from each mussel and fixed in Davidson’s solution for at least 72 h. After fixation, the samples were dehydrated in alcohol, cleared in xylene, and embedded in paraffin wax. Sections of 4 μm in thickness were cut in a Thermo Scientific microtome, dried at 37 °C for 24 h, and stored at room temperature; the sections were stained with hematoxylin and eosin (H&E). Gross examinations were performed under a stereomicroscope (Nikon SMZ 800). The specimens were examined under a Zeiss Axioscope A1 light microscope. Ziehl-Neelsen staining was also performed to detect Nocardia (acid-fast bacterium) [18].

## 3. Statistical Analysis

McNemar’s test for two related binomial proportions (conditional) was used to evaluate the agreement between the two tests performed on the same animal. Statistical significance was set at *p* < 0.05.

## 4. Results

Supplemental Figure S2 shows a geographical map of the Campania Region’s coastline with the locations of the marine sites where all mussel samples were collected. The coordinates of the marine sites are shown in Supplemental Table S1. ddPCR detected *N. crassostreae* DNA in ~26% of the pooled samples (48/185) and ~15% of the individual samples (23/149). qPCR revealed *N. crassostreae* DNA in ~3% (6/185) of the pooled samples and ~4% (6/149) of the individual samples (Table 1).

The ddPCR method detected more pathogen DNA than the qPCR method for both sets of mussel samples. Significant differences between the two molecular assays for detecting *N. crassostreae* DNA were found in both the pooled and individual samples (*p* < 0.05). 

DNA copies/microliter ranged from <1 to 11 and <1 to 3.54 in pooled and individual samples, respectively. The fluorescence amplitude of the droplets was used to calculate the number of DNA copies/microliter (Supplemental Figure S3A,B).

PCR analysis detected amplicons, the sequencing of which showed 100% identity with the *N. crassostreae* 16S–23S rRNA intergenic spacer sequences deposited in GenBank (Accession Number AF536419.1).

**Figure 1:**
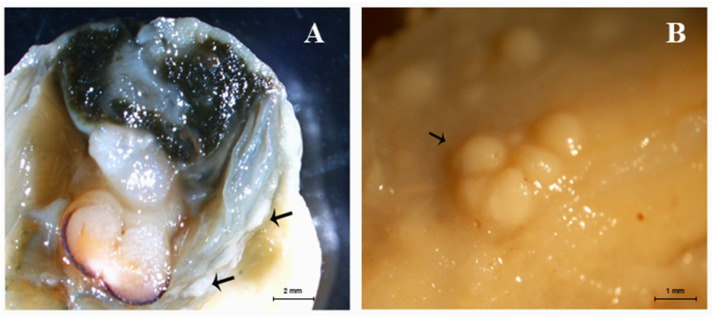
Nodular proliferation on gills (**A**) and mantle (**B**) of affected mussels (black arrows).

**Figure 2:**
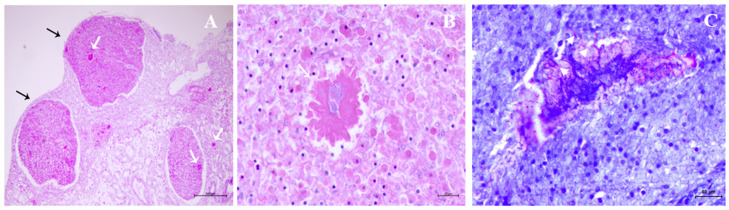
(**A**) *N. crassostreae* colonies (white arrows) in the abscess-like nodules of mantle. Black arrows indicate residual constitutive tissue. (**B**) *N. crassostreae* colonies at higher magnification. Hematoxylin and eosin staining. (**C**) Filamentous structures, typical morphology of *Nocardia* spp., are shown by Ziehl-Neelsen staining (arrow heads).

In the diseased mussels, macroscopic tissue damage caused by *N. crassostreae* was characterized by scattered nodules prevalent at the gill and mantle levels (Figure 1A,B). Microscopic findings of nodular proliferation were characterized by the presence of *Nocardia* colonies delimited by partly degenerated hemocytes, the immune-effector cells of mollusks. These nodules shared some resemblance with purulent collections from higher vertebrates (Figure 2A,B). In these individual samples, *N. crassostreae* DNA ranged from 0.3 to 1.06 copies/μL by ddPCR.

## 5. Discussion

The morphological and molecular findings of the current study were consistent with *N. crassostreae* infection. This is the first systematic study carried out on *Nocardia* using ddPCR as a diagnostic procedure. Our findings showed that ddPCR is very useful for identifying and quantifying *N. crassostreae* DNA in mollusks destined for human consumption and is able to accurately diagnose *N. crassostreae* infection. Therefore, ddPCR outperformed real-time qPCR for *N. crassostreae* DNA detection, confirming that ddPCR technology is able to identify pathogens in many infectious diseases with higher sensitivity and specificity [12].

ddPCR investigation performed on pooled mussel samples allowed us to gain insights into the spreading of *N. crassostreae* infection in mussels, thereby showing it to be an important, major concern for safe food that should not be underestimated. Furthermore, the molecular and histological findings observed in individual mussels affected by morphological tissue damage caused by *N. crassostreae* allowed suggesting that bacterium copy numbers found in these mollusks can give valuable insights into the necessary infectious dose. We found 0.3 copy numbers/μL in samples of mussels showing histological lesions by *N. crassostreae*, which suggests that these copy numbers are enough to be considered potentially infectious and able to cause bacterial disease in mollusks. It is worth noting that the infectious dose of pathogenic *Nocardia* species in human hosts is unknown [9].

Recently, it has been suggested that bivalve microbiomes can provide a model to better understand human–microbe interactions, particularly within the context of host diseases and environmental change [19]. Our investigation showed that *N. crassostreae* can be an emerging pathogen that is widespread in mussels, thus representing a biological hazard for human health, since *N. crassostreae* has been reported as a human pathogen both in immunocompromised and immunocompetent individuals [7,8]. Aquaculture currently accounts for approximately 52% of the total quantity of fish and shellfish consumed by humans. Its annual production continues to expand. In particular, seventeen million tons of marine bivalve mollusks were produced in 2018 [20]. 

The hazards associated with human pathogenic bacteria in products from aquaculture can be from indigenous bacteria, which are naturally present in the aquatic environment, and bacteria from anthropogenic contamination [20]. Shellfish-growing areas are increasingly affected by anthropogenic impacts, such as the release of agricultural run-off, untreated sewage effluents at outfall sites, and effluents of wastewater treatment plants, which may function as reservoirs of bacterial agents in marine environments [21,22]. It has been suggested that environmental pressures placed on marine habitats result in microbial dysbiosis, which is a key event in diseases in marine ecosystems [23]. Nocardiosis is emerging as an important disease of cultured and freshwater fishes. Several *Nocardia* spp. including *N. seriolae*, *N. crassostreae*, *N. salmonicida*, *N. asteroides*, and *N. xestospongieae* have been isolated from fish and shellfish, thus representing economically important pathogens in aquaculture [24,25]. Additional cases of nocardiosis have recently been reported in marine organisms. For the first time, *N. otitidiscaviarum* and *N. farcinica* associated with severe tissue damage have been described in free-ranging cetaceans [26].

The microbial communities of mollusks include both resident (or core) and transient microbiota, and long-standing evidence supports the importance of maintaining healthy populations of microbiota for the survival, homeostasis, and complete development of marine mollusks [27]. Bivalve mollusks are highly susceptible to pathogen introduction and chemical pollutants in coastal marine environments from anthropogenic activities, which dramatically alter the mollusk microbiota, affecting the ability of beneficial mollusk microbes to resist both known and unknown bacterial pathogens, thus allowing the subsequent colonization of pathogenic *Nocardia* bacteria [27]. Mussels feed by filtering seawater and, thus, are able to accumulate pathogenic microorganisms naturally occurring in the water in which they live. In particular, *N. crassostreae* is believed to be an emerging pathogen of molluscan bivalves, which warrants systematic and globalized monitoring, as it shows zoonotic potential [28].

The food safety risk from this pathogen due to the consumption of aquaculture products is still poorly understood [29]. The anatomo-clinical, laboratory, and epidemiological data on nocardiosis are very limited in the medical literature, and most of our knowledge about *Nocardia* spp. infections is based on case reports, which makes their significance in human and veterinary medicine overlooked and misunderstood, although *Nocardia* spp. can cause life-threatening infections [2,30]. Aquaculture is predicted to supply the majority of aquatic dietary protein by 2025 [31]. However, its impact, including early evaluation of unavoidable risks for human health, wildlife welfare, and environmental integrity, is basically unknown [32]. Therefore, future efforts in aquaculture systems should be focused on the One Health framework to describe the impact of pathogens on the environment, wildlife, and human conditions and set practical metrics that the industry has to follow to gain sustainability and achieve beneficial health for people, nonhuman organisms, and their shared environment [31,32].

## Figures and Tables

**Table 1:** This table provides the results obtained by comparing the ddPCR and qPCR methods (N = number of samples).

Sample Set	N	ddPCR	qPCR
Pooled tissues	185	26%	3%
Individual mussels	149	15%	4%

## Data Availability

The datasets presented in this study can be found in online repositories. The names of the repository/repositories and accession number(s) can be found in the article/Supplementary Material.

## Supplementary Materials

The following Supporting Information can be downloaded at: https://www.mdpi.com/article/10.3390/pathogens12080994/s1, Figure S1: The ddPCR set up and development for the detection and quantification of *Nocardia crassostreae* is represented in this workflow. Figure S2: Geographical map showing marine sites utilized for mussels growing. All sites were located alongside the coastline of Regione Campania in the Mediterranean Sea (Tyrrhenian Sea). Figure S3: Rain plots of ddPCR for *N. crassostreae* DNA detection. Blue droplets = droplets positive; gray droplets = droplets negative for *N. crassostreae* DNA. (A). Positive droplets for *N. crassostreae* DNA in individual mussel samples with an amplitude ranging from 1500–7500; (B). Positive droplets for *N. crassostreae* DNA detected in pooled mussel samples with an amplitude between 1500 and 4500. Table S1: This table summarizes the coordinates of the all marine sites where mussel samples were collected from.
