# Supplementary material for: Detection and Quantification of Nocardia crassostreae, an Emerging Pathogen, in Mytilus galloprovincialis in the Mediterranean Sea Using Droplet Digital PCR
Source: Pathogens. 2023 Jul 28;12(8):994. doi: 10.3390/pathogens12080994 (PMC10458358; doi:10.3390/pathogens12080994)
Supplement: Supplementary file 1 [file pathogens-12-00994-s001.zip › supplemental Table S1.pdf]

Table S1. This table summarizes the coordinates of the all marine sites where mussel samples were collected from.

	**SITES**	**COORDINATES**
1	CASTELLAMMARE DI STABIA	40°41'45"N-14°28'32"E
2	MONTE DI PROCIDA	40°47'41"N-14°02'37"E
3	TORRE DEL GRECO	40°46'17"N-14°22'58"E
4	TORRE DEL GRECO	40°47'01"N-14°21'38"E
5	GIUGLIANO IN CAMPANIA	40°53'15"N-14°02'19"E
6	LAGO FUSARO	40°49'25"N-14°03'18"E
7	NISIDA	40°47'39"N-14°09'37"E
8	RADA SANTA LUCIA	40°49'52"N-14°15'04"E
9	TORRE ANNUNZIATA	40°45'15"N-14°26'06"E
10	CAPO MISENO	40°46'53"N-14°05'16"E
11	POZZUOLI	40°49'45"N-14°04'48"E
12	BACOLI	40°47'55"N-14°05'04"E
13	ERCOLANO	40°48'11"N-14°20'27"E
14	CASTEL DELL'OVO	40°49'44"N-14°14'50"E
15	BAGNOLI	40°48'53"N-14°09'40"E
