# Supplementary figures and images for: Detection and Quantification of Nocardia crassostreae, an Emerging Pathogen, in Mytilus galloprovincialis in the Mediterranean Sea Using Droplet Digital PCR
Source: Pathogens. 2023 Jul 28;12(8):994. doi: 10.3390/pathogens12080994 (PMC10458358; doi:10.3390/pathogens12080994)

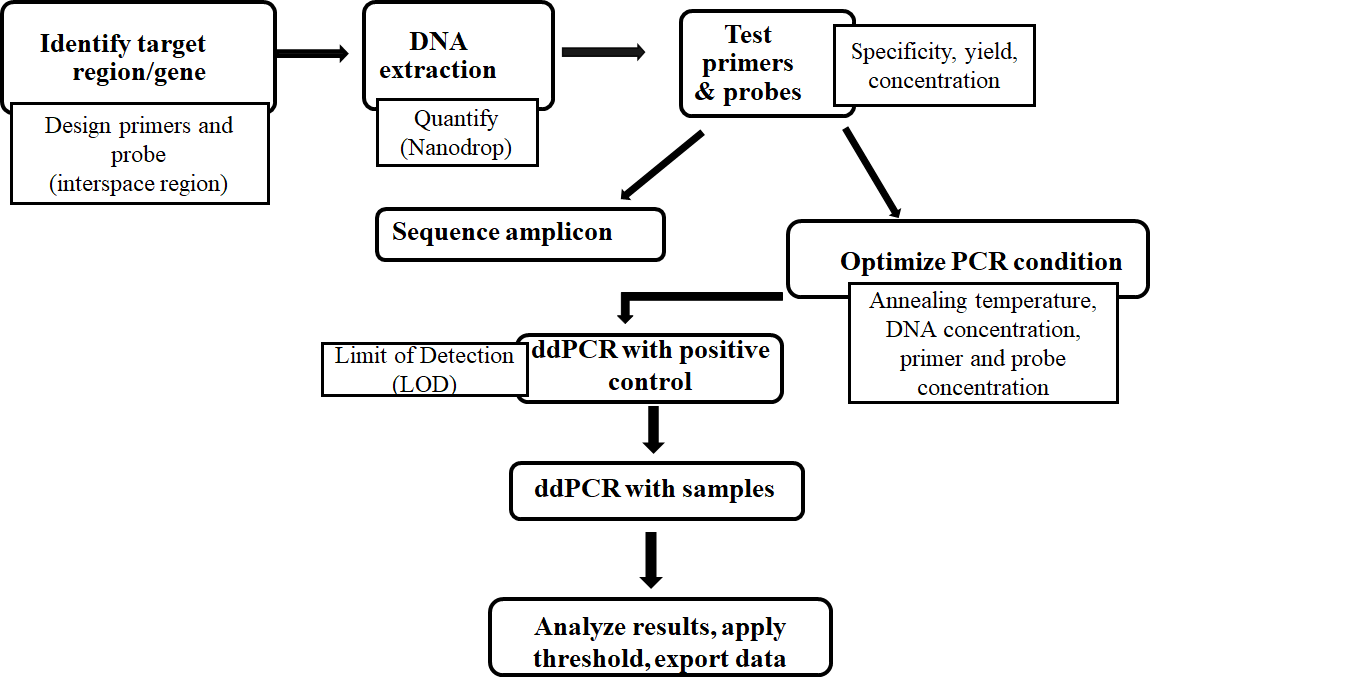

Supplement: Supplementary file 1 [file pathogens-12-00994-s001.zip › Figure S1.tif]

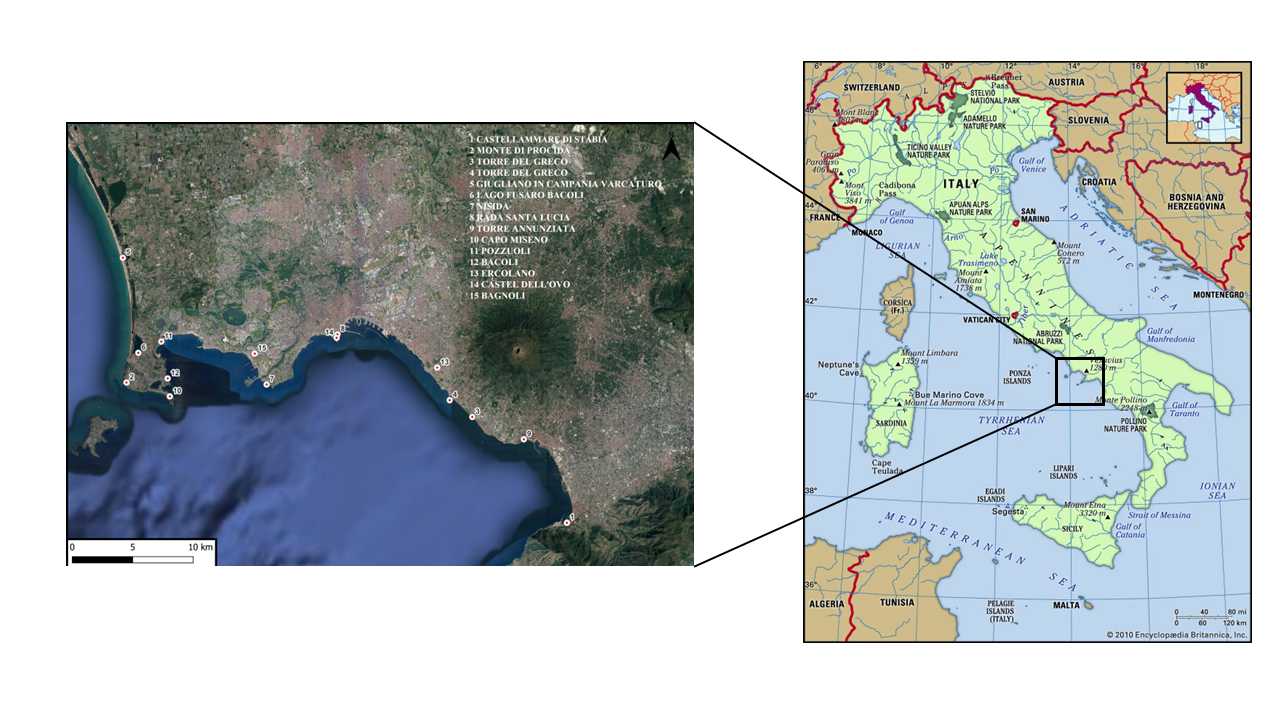

Supplement: Supplementary file 1 [file pathogens-12-00994-s001.zip › Figure S2.TIF]

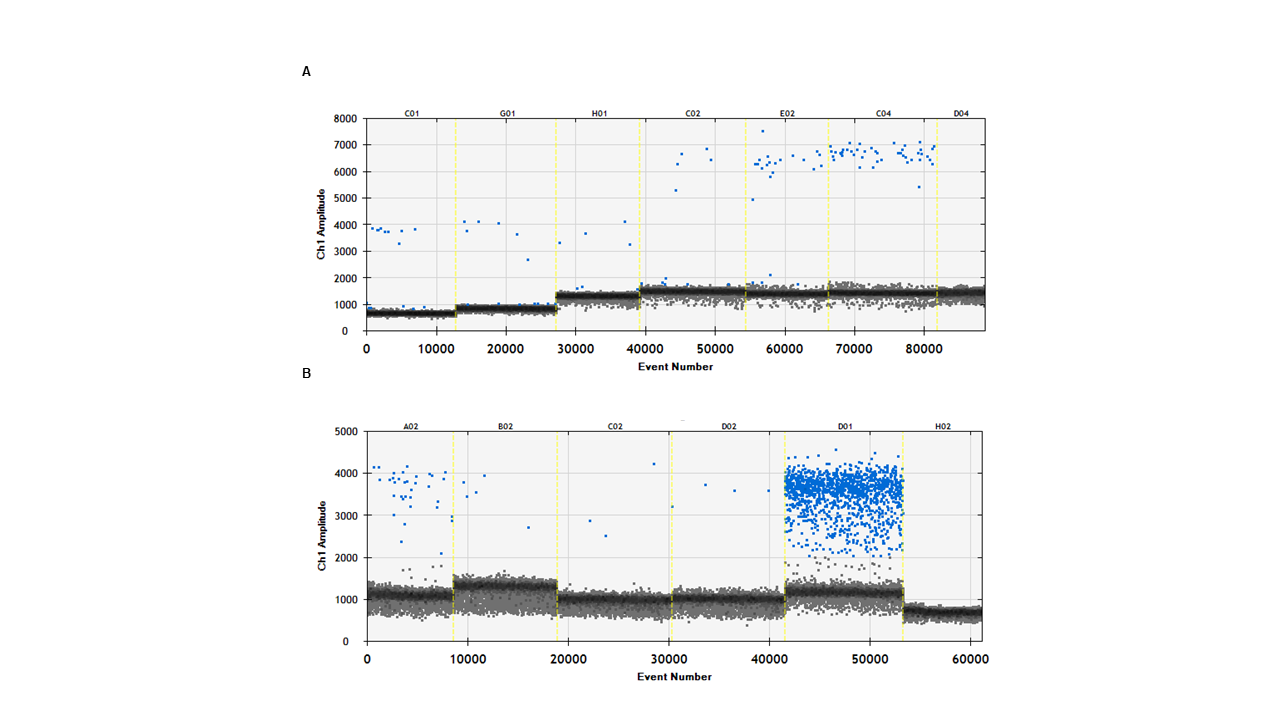

Supplement: Supplementary file 1 [file pathogens-12-00994-s001.zip › Figure S3.TIF]
